# Screening natural product extracts for potential enzyme inhibitors: protocols, and the standardisation of the usage of blanks in α-amylase, α-glucosidase and lipase assays

**DOI:** 10.1186/s13007-020-00702-5

**Published:** 2021-01-06

**Authors:** Chintha Lankatillake, Shiqi Luo, Matthew Flavel, George Binh Lenon, Harsharn Gill, Tien Huynh, Daniel Anthony Dias

**Affiliations:** 1 School of Health and Biomedical Sciences, Discipline of Laboratory Medicine, RMIT University, Bundoora, 3083 Australia; 2 TPM Bioactives Division, The Product Makers Pty Ltd, Melbourne, Australia; 3 School of Life Sciences, La Trobe University, Melbourne, Australia; 4 School of Science, RMIT University, Bundoora, 3083 Australia

**Keywords:** α-amylase, α-glucosidase, Blanks, Enzyme inhibition, Lipase, Methodology, Natural products, Plant extracts, Protocol, Standardisation

## Abstract

**Background:**

Enzyme assays have widespread applications in drug discovery from plants to natural products. The appropriate use of blanks in enzyme assays is important for assay baseline-correction, and the correction of false signals associated with background matrix interferences. However, the blank-correction procedures reported in published literature are highly inconsistent. We investigated the influence of using different types of blanks on the final calculated activity/inhibition results for three enzymes of significance in diabetes and obesity; α-glucosidase, α-amylase, and lipase. This is the first study to examine how different blank-correcting methods affect enzyme assay results. Although assays targeting the above enzymes are common in the literature, there is a scarcity of detailed published protocols. Therefore, we have provided comprehensive, step-by-step protocols for α-glucosidase-, α-amylase- and lipase-inhibition assays that can be performed in 96-well format in a simple, fast, and resource-efficient manner with clear instructions for blank-correction and calculation of results.

**Results:**

In the three assays analysed here, using only a buffer blank underestimated the enzyme inhibitory potential of the test sample. In the absorbance-based α-glucosidase assay, enzyme inhibition was underestimated when a sample blank was omitted for the coloured plant extracts. Similarly, in the fluorescence-based α-amylase and lipase assays, enzyme inhibition was underestimated when a substrate blank was omitted. For all three assays, method six [**R**aw **D**ata - (**Su**bstrate + **Sa**mple **B**lank)] enabled the correction of interferences due to the buffer, sample, and substrate without double-blanking, and eliminated the need to add substrate to each sample blank.

**Conclusion:**

The choice of blanks and blank-correction methods contribute to the variability of assay results and the likelihood of underestimating the enzyme inhibitory potential of a test sample. This highlights the importance of standardising the use of blanks and the reporting of blank-correction procedures in published studies in order to ensure the accuracy and reproducibility of results, and avoid overlooked opportunities in drug discovery research due to inadvertent underestimation of enzyme inhibitory potential of test samples resulting from unsuitable blank-correction. Based on our assessments, we recommend method six [RD − (Su + SaB)] as a suitable method for blank-correction of raw data in enzyme assays.

## Background

Enzymes are the molecular targets of almost half of all marketed small-molecule drugs [1, 2]. Their protein structure affords a high level of druggability and target validation which makes enzymes an attractive target for novel drug discovery efforts [3]. Enzyme inhibitors form an important class of clinical drugs ranging in use from cancer [4, 5], cardiovascular disease [6, 7], diabetes [8, 9], neurological disorders [10–13] and obesity [14, 15]. Inhibitors of the endogenous carbohydrases α-glucosidase and α-amylase, reduces postprandial hyperglycaemia by delaying the digestion of dietary carbohydrates and are valuable therapeutics in the management of diabetes [8, 16]. Similarly, calorie restriction imposed by inhibition of carbohydrases and pancreatic lipase is useful for the prevention of weight gain and the treatment of obesity [14, 17]. Therefore, enzyme inhibition assays targeting α-glucosidase, α-amylase and lipase are widespread in research, and screening plant extracts and natural products for inhibitory activity against these enzymes is a common approach for the discovery of potential antidiabetic and antiobesity drugs to treat and manages these metabolic diseases [18–20].

Natural products are secondary metabolites produced by living organisms such as plants and microorganisms [21]. An abundant and easily accessible source of natural products is the kingdom of plants, a large proportion of which remains to be explored for potential bioactive metabolites [21]. These molecules possess high chemical and structural diversity unrivalled by synthetic compound libraries [22–24], have evolved intrinsic bioactive properties due to their evolutionary biological roles in living organisms [23–26], and are excluded from Lipinski’s rules of five [23, 24]. Therefore, natural products are an attractive source of therapeutic molecules and a significant body of research is devoted to the discovery of drugs from natural product extracts such as plant extracts [23, 24].

**Fig. 1:**
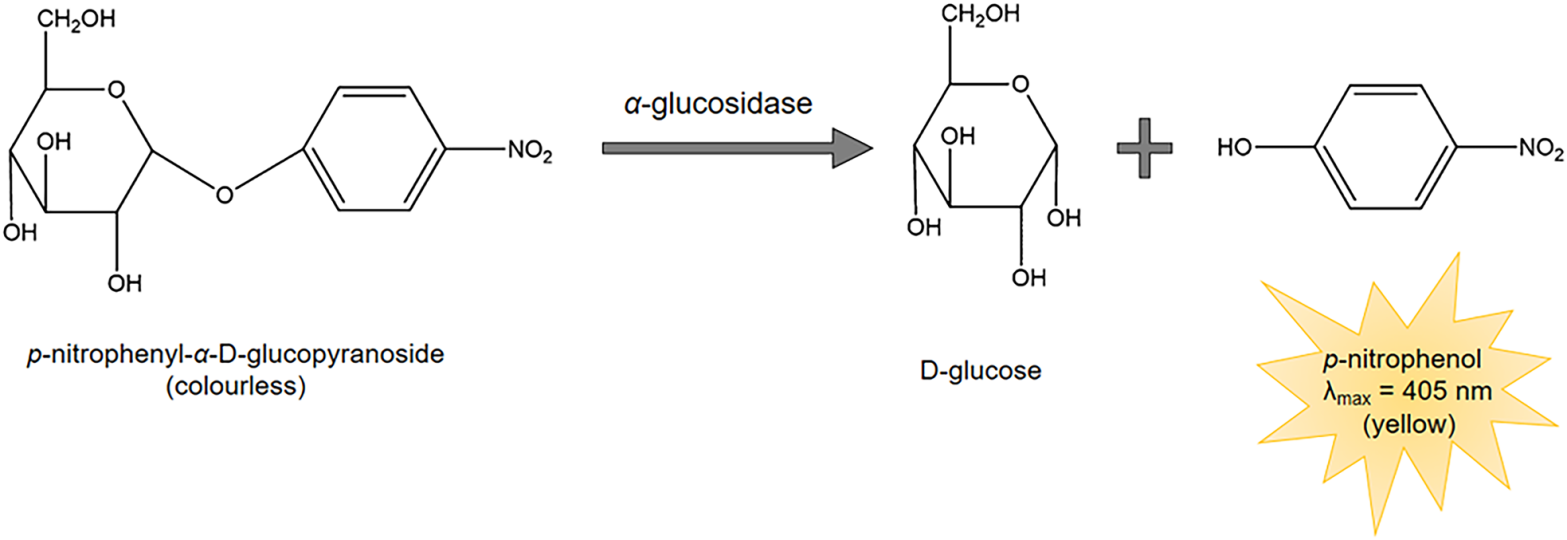
Hydrolysis of colourless *p*-nitrophenyl-α-d-glucopyranoside to coloured *p*-nitrophenol by α-glucosidase Adapted from [27]

In-lab approaches for screening enzyme inhibitors from natural product extracts are based on spectroscopy and often determines enzyme activity using specially designed, labelled substrates which, upon enzymatic cleavage, produce a spectrometrically measurable signal either as colour (absorbance) or fluorescence of a defined wavelength [27–31]. For example, the chromogenic substrate *p*-nitrophenyl-*α*-d-glucopyranoside (*p*NPG) is widely used for the determination of α-glucosidase activity (Fig. 1). *p*NPG is a colourless molecule which contains a d-glucose residue linked to a *p*-nitrophenol moiety via a glycosidic bond. Hydrolysis of this glycosidic bond by α-glucosidase releases *p*-nitrophenol (coloured product), enabling the spectrophotometric determination of α-glucosidase activity at the absorbance maxima of *p*NPG; λ = 405 nm [27, 32].

**Fig. 2:**
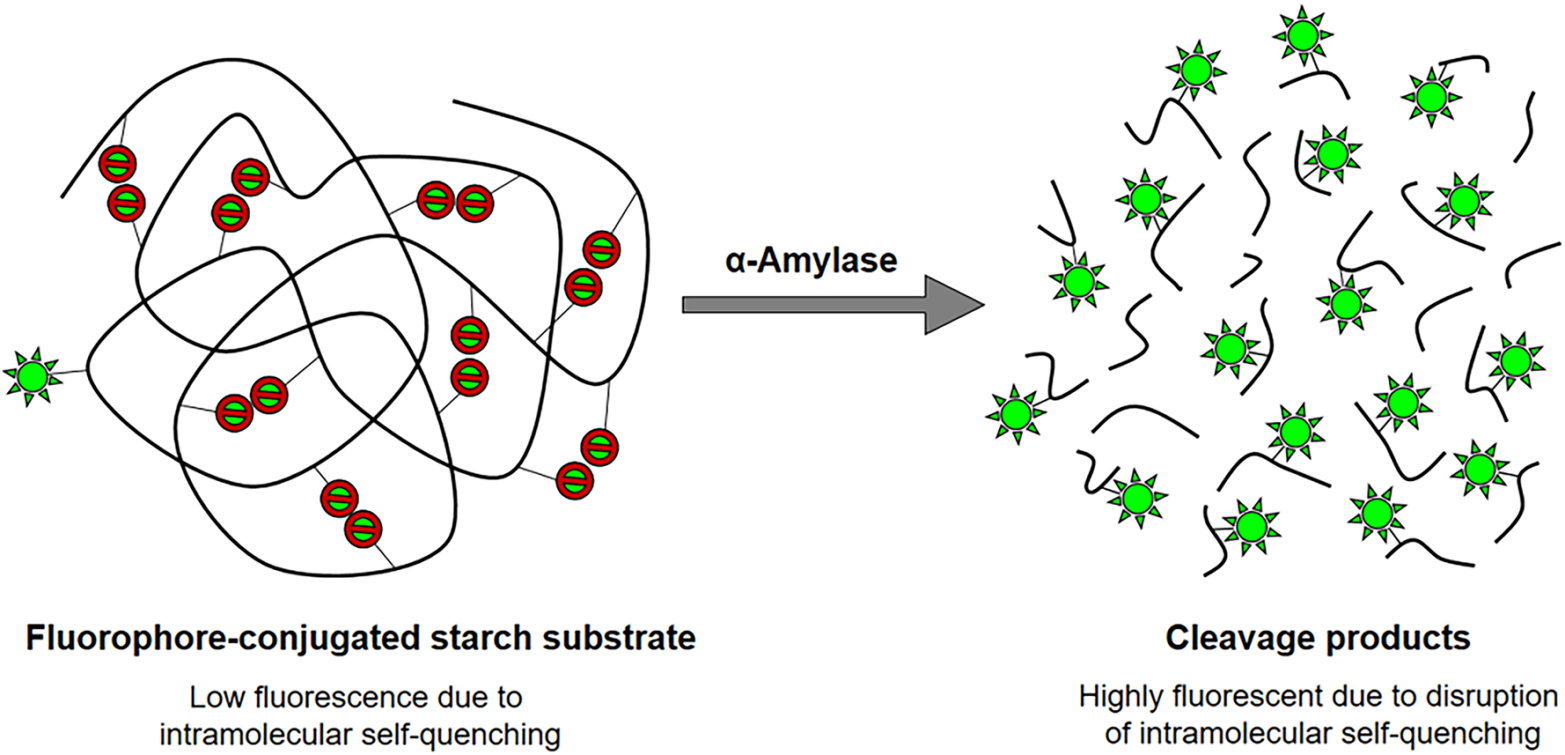
Schematic illustration of the BODIPY^®^FL-DQ™ starch assay concept Adapted from [36]

Alternatively, there are also fluorescence-based enzyme assays which make use of substrates linked to a fluorophore. Enzymatic cleavage releases the fluorophore which re-emits light (fluoresces) upon excitation at a specific wavelength [33–35]. A routinely used substrate for fluorescence-based α-amylase assays is the BODIPY^®^ FL-DQ™ (boron-dipyrromethene fluorescent dye quenching) starch which consists of a starch derivative (DQ™ starch) conjugated to the green fluorescent BODIPY^®^ FL fluorophore. The DQ™ starch is heavily labelled with BODIPY^®^ FL to such an extent that the close proximity of the fluorophores to each other results in intramolecular self-quenching and the substrate fluorescence is almost completely quenched (Fig. 2). As the substrate is hydrolysed by α-amylase, the intramolecular self-quenching is disrupted causing an intense increase in green fluorescence [28, 36].

**Fig. 3:**
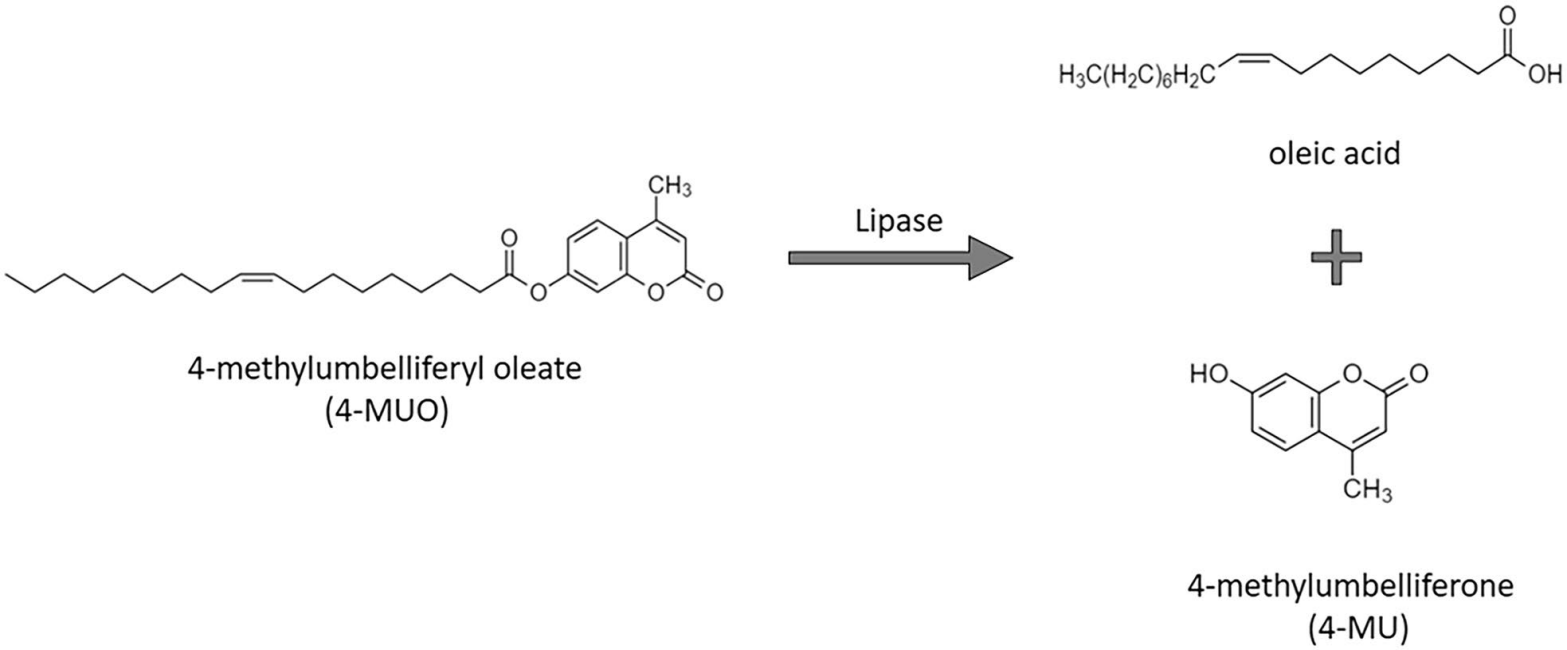
Hydrolysis of 4-MUO by lipase Modified from [39]

A commonly used fluorogenic substrate for lipase assays is 4-methylumbelliferyl oleate (4-MUO) [37]. Lipase-catalysed cleavage of 4-MUO liberates the fluorescent product, 4-methylumbelliferone (4-MU) (Fig. 3), in proportion to lipase activity [37–39].

These enzyme assays can be performed in 96-well microplate readers [40–42], or in traditional cuvette-based spectrophotometers or spectrofluorometers [43–45]. However, miniaturisation of assays by adapting them to a 96-well format is beneficial as it reduces the volumes of reagents required, is cost-efficient and enables faster testing suitable for screening large libraries of plants, extracts or natural products [46].

Spectrometry-based assays are subject to spectral interference from sample colour, autofluorescence, and turbidity arising from poor solubility [47, 48]. Therefore, these factors must be taken into consideration when designing and carrying out enzyme-based assays with natural product extracts.

Plant extracts are often highly coloured as they contain natural, highly conjugated pigments. The colouration of plant extracts and natural products can cause interference with absorbance measurements in spectrophotometric assays [47]. Chlorophylls and carotenoids are the two major classes of photosynthetic plant pigments. Chlorophylls absorb light strongly in the 430–480 nm and 640–660 nm range [49]. However, chlorophylls are also capable of absorbing light at other wavelengths. Carotenoids encompass a wide range of compounds such as *β*-carotene, phytoene and lycopene, and have a wide visible light absorbance spectrum ranging from 400–530 nm [50]. Betalains, which includes betacyanins and betaxanthins [51]; and flavonoids such as anthocyanins and flavanols [52, 53] are the other classes of pigments contributing to colour in plants. All these phytochemical classes can interfere with assays which use wavelengths that overlap their absorption spectra.

In addition to the pigments intrinsic to plants, post-harvest changes can lead to the development of additional coloured products. For example, the action of polyphenol oxidases generates melanins and other brown pigments in harvested plant tissue exposed to oxygen. This process, referred to as enzymatic browning, is particularly common in fruits [54–56]. Samples with high sugar content, such as sugarcane (*Saccharum* spp.) extracts, are susceptible to intense browning caused by the Maillard reaction and caramelisation reactions [57]. The coloured products of such post-harvest reactions can be a significant source of interference in absorbance-based assays.

Autofluorescence is observed in some plants (*e.g. Berberis vulgaris* L.*, Humulus lupulus* L.*, Matricaria chamomilla* L., and *Salvia officinalis* L. [28]) and endogenous natural products [58–61] in a range of wavelengths which can interfere with fluorescence-based assays. For instance, anthranilates, alkaloids, coumarins, and stilbenes fluoresce in the blue-violet range (~ 400–520 nm), flavones and flavonoids in the green-yellow range (~ 520–590 nm), polycyclic aromatic quinones, tannins and some alkaloids in the orange range (~ 635–590 nm), and chlorophyll, porphyrins and certain quinones fluoresce in the red-far red range (~ 590–700 nm) [59, 60].

Poor solubility of some extracts and compounds in the assay buffers results in turbidity due to the presence of undissolved, suspended particles and may lead to inaccurate results. Light passing through a turbid medium is subject to multiple scattering and absorption events [62]. Therefore, turbidity interferes with spectrophotometric measurements by increasing absorbance and can result in misleadingly high readings [63]. Similarly, the absorbance and scattering of photons in a turbid medium can also distort fluorescence measurements [62].

The substrate can also be a source of error in enzyme assays. For example, unstable substrates may gradually decay to form their product. Contamination of the substrate with the chromogenic or fluorogenic product introduces a false signal and can cause a misleading increase in absorbance or fluorescence which is unrelated to enzyme activity [64].

In summary, assay interference due to sample colour, autofluorescence and turbidity can contribute to errors in measurements and hence affect the accuracy and reproducibility of results [47, 63]. Therefore, it is essential to minimise the effects of these interferences by blank-correcting raw data (RD) using appropriate sample and reagent blanks.

A sample blank contains an equal concentration of the test sample—whether it be an extract, an isolated compound, or a drug used as a control—without the enzyme or substrate. The absorbance (or fluorescence) of the sample blank quantifies the absorbance (or fluorescence) contributed by the colour, autofluorescence and/or turbidity of the sample. Subtracting the sample blank reading from the test well (which contains the enzyme + substrate + test sample) reading provides the value of the absorbance or fluorescence which is due to the enzymatic reaction; i.e. the *‘true’* value contributed by the reaction product.

The optical properties of different test samples vary widely. Therefore, to ensure the accuracy of results, it is necessary to include a sample blank for *each* sample in multi-sample assays, a *“positive control blank”* (equivalent to a sample blank for the positive control), and a *“negative control blank”* (equivalent to a sample blank for the negative control), and to correct each measurement using their respective blanks. Sample blanks have been included in some published studies [45, 65, 66], however, many appear to overlook the use of a sample blank in their experiments.

A practical approach to minimise assay interference from substrate contamination and degradation is to use a reagent (substrate) blank [63]. Some published studies [65, 67] have included a substrate blank to eliminate any false signals due to colour (absorbance) or fluorescence of the substrate. However, as with the sample blanks, many studies appear to omit the use of a substrate blank. Instead, many report using either a buffer blank or a blank that is not defined in the publications.

Although the use of blanks in experiments is common practice, there is currently no consensus on which blanks should be used. Published studies vary widely with regards to which blanks are included for the calculation of blank-corrected data. Researchers have previously reported assays using a buffer blank [68–70], sample blank [66, 71–74], a substrate blank [75, 76], or even an enzyme blank [77, 78]. Some researchers have combined substrate and sample in a single blank to account for interference from both [65, 79, 80]. Therefore, there appears to be many ways of blank-correction, with no standardisation, which not only adds to the confusion of researchers attempting these enzyme inhibition assays, it also adds bias and makes it difficult to compare results between studies. There are no studies that have investigated whether the type of blank used influences the calculated enzyme activity or enzyme inhibition results, which makes the selection of appropriate blanks problematic. In addition, despite the availability of a range of assay methods and countless publications involving α-glucosidase, α-amylase, and lipase inhibition assays, there is a lack of comprehensive published protocols that detail the assays *step*-*by*-*step*.

The aims of this study were to (1) provide comprehensive protocols for carrying out a colorimetric (absorbance) α-glucosidase inhibition assay, and fluorometric α-amylase and lipase inhibition assays in 96-well microplates; and (2) investigate whether using a buffer blank, substrate blank, sample blank, or a combination of blanks for blank-correction will affect the final, calculated enzyme inhibition results.

## Materials and methods

### Plant samples

Six medicinal plants with evidence of enzyme inhibitory activity against α-glucosidase (*Aegle marmelos* (L.) Corrêa [81, 82] and *Phyllanthus niruri* L. [83–86]), α-amylase (*Gardenia jasminoides* Ellis. [87] and *Nelumbo nucifera* Gaertn [87]), and lipase (*Camellia sinensis* L. [14, 88–90] and *Sophora japonica* L. [14]) were selected for the enzyme assays, based on the literature, to determine how their pigmentation and the use of different blanks and blanking methods affect the final assessment of their bioactivity. These medicinal plants have previously been investigated by our research group for their antidiabetic and antiobesity properties. They were chosen for initial testing based on a systematic review conducted on promising bioactive plants which highlighted the above species. These plants were selected for inclusion in this study because their ability to inhibit the respective enzymes have already been established in studies published by others [81–86, 88–90] and in previous studies carried out by our research group [14, 87].

*Aegle marmelos* was identified by local experts and dry leaves were collected in Kandy, Sri Lanka on 30/05/2017 by Dr Tien Huynh. Dried *P. niruri* leaf samples were obtained from a commercial herbal products supplier in Malaysia (identified and collected by the supplier in Jalan Jelebu, Malaysia on 20/02/2017). Herbarium samples can be found at the Janaki Ammal Herbarium, Indian Institute of Integrative Medicine (Accession No. 18696) for *A. marmelos* and the University of South Florida Herbarium, Institute for Systematic Botany (Accession No. 66964) for *P. niruri*.

Commercially prepared herbal extract granules (Nong’s, HK) of *S. japonica* flowers (Batch No. A1601428; 1203A1601428071119), *N. nucifera* leaves (Batch No. A1601450; 1356A1601450060919), *G. jasminoides* fruit (Batch No. A1600810; 1033A1600810050719) and *C. sinensis* (matcha) leaves were provided by GL Natural Healthcare Clinic, Strathmore, Victoria, Australia.

### Positive controls

Acarbose, a known pharmacological inhibitor of α-glucosidase and pancreatic α-amylase was used as the positive control in the α-glucosidase and α-amylase assays [45, 67, 91]. Orlistat, a known inhibitor of pancreatic lipase was used as the positive control in the lipase assay [92, 93]. Acarbose (Cat. No. ACR459080010), and orlistat (Cat. No. O4139) were purchased from Thermo Fisher Scientific Australia and Sigma-Aldrich, respectively.

### Chemicals and reagents

Analytical grade ethanol (Cat. No. 111727), and dimethyl sulfoxide (DMSO) (Cat. No. 102952) were purchased from Merck Millipore Australia. The EnzChek™ *Ultra* Amylase assay kit (Cat. No. E33651) was purchased from Life Technologies Australia, α-amylase from porcine pancreas (EC Number 3.2.1.1, Cat. No. 10102814001), lipase from porcine pancreas (Type VI-S, EC Number 3.1.1.3, Cat. No. L0382), 4-methylumbelliferyl oleate (4-MUO) (Cat. No.75164), Tris base (Cat. No. 10708976001), intestinal acetone powders from rat (Cat. No. I1630), calcium chloride (CaCl_2_), and sodium chloride (NaCl) were purchased from Sigma-Aldrich Australia. *p*-nitrophenyl α-d-glucopyranoside (pNPG) (Cat. No. ACR337150050), and phosphate-buffered saline (PBS) tablets (Gibco) (Cat. No. 18912014), were purchased from Thermo Fisher Scientific Australia. Anhydrous disodium hydrogen phosphate (Na_2_HPO_4_, AnalaR^®^), and sodium dihydrogen phosphate dihydrate monobasic (NaH_2_PO_4_.H_2_O, UNIVAR^®^) were obtained from AJAX Chemicals Australia and BDH Chemicals Australia, respectively. Milli-Q^®^ water was obtained from a Millipore Milli-Q^®^ water purification system. Distilled water was used in the preparation of buffers and reagents unless otherwise stated.

Corning^®^ Costar^®^ tissue culture-treated clear, flat bottom 96-well plates (Cat. No. CLS3516) were purchased from Sigma-Aldrich Australia. Nunc™ Nunclon™ delta treated, black polystyrene, flat bottom 96-well plates (Cat. No. 137101) were purchased from Thermo Fisher Scientific Australia.

### Methods

An absorbance assay with *p*NPG as the substrate was used for the determination of α-glucosidase activity, and fluorescence assays using the substrates BODIPY^®^FL-DQ™ starch, and 4-MUO were used for the determination of α-amylase and lipase activity, respectively. Acarbose was the positive control in the α-glucosidase and α-amylase assays while orlistat was the positive control in the lipase assay. The corresponding assay buffer was used as the negative control in each of the assays.

#### Absorbance assay: *α*-glucosidase

The α-glucosidase assay was adapted from [91, 93] with modifications. The method is described in detail in the proceeding section “α-glucosidase assay step-by-step.”

##### Extraction of plant samples

The leaves of *Aegle marmelos* and *Phyllanthus niruri* were separated from the dried plant samples, homogenized to a fine powder using a mortar and pestle or an electric blender (KitchenAid^®^), and the homogenized samples were stored in the dark at room temperature (RT = 25 °C) with silica gel desiccant.

Approximately 50 mg of the powdered leaf samples were further homogenized with 500 µL of 100% ethanol in lysing tubes using an MP Biomedicals™ FastPrep-24™ instrument. Each sample was then extracted for 15 min at 37 °C and 900 rpm using an Eppendorf ThermoMixer^®^, and then centrifuged for 15 min at 12,700 rpm at RT. The supernatant (ethanol extract) was subsequently transferred into a new Eppendorf tube. The pellet was re-suspended in 500 µL of Milli-*Q* water and allowed to extract for 15 min at 37 °C and 900 rpm using an Eppendorf ThermoMixer^®^. The supernatant (water extract) was then combined with the ethanol extract that was previously generated. The combined ethanol/water extract (50:50) was centrifuged for 10 min at 12,700 rpm at RT until a clear supernatant was obtained. The extracts were concentrated by evaporating solvents under reduced pressure using a rotational vacuum concentrator (Martin Christ RVC 2-33 CDplus) operating at 1500 rpm at 30 °C for 6–8 h. The average yields of the concentrated extracts were 0.3 mg for *A. marmelos* and 0.8 mg for *P. niruri*. The concentrated extracts were stored in darkness at RT with silica gel desiccant until required.

##### Preparation of buffers, reagent solutions, test samples and controls

- *α-glucosidase assay buffer I* (for preparing extracts and acarbose) consisted of freshly prepared 100 mM sodium phosphate buffer with 2% DMSO (pH 6.9). All test extracts and acarbose (positive control) were prepared by dissolving in assay buffer I.
- Comments: DMSO was used to aid in the solubility of the extracts in the buffer [94, 95]. Preliminary testing confirmed that 2% DMSO (final concentration of 0.001% DMSO) had no significant effect on rat intestinal α-glucosidase activity. It is also possible to use PBS (pH adjusted to 6.9) as the incubation buffer in this assay without affecting rat intestinal α-glucosidase activity.
- *α-glucosidase assay buffer II* (for preparing assay reagents) consisted of a freshly prepared 100 mM sodium phosphate buffer without DMSO (pH 6.9). The enzyme and substrate solutions were prepared by dissolution in assay buffer II.
- *Test samples* were prepared from the concentrated plant extracts by dissolving and diluting the extracts in α-glucosidase assay buffer I to obtain working test sample solutions of 1 mg/mL. These were freshly prepared immediately before experiments and used on the same day.
- *Positive control, acarbose* powder was dissolved in α-glucosidase assay buffer I to prepare a 100 mg/mL stock solution which was stored short-term at 4 °C. A working solution of 1 mg/mL acarbose was prepared by diluting the stock solution in α-glucosidase assay buffer I immediately before experiments.
- *Negative control* (vehicle) was α-glucosidase assay buffer I.
- *Rat intestinal α-glucosidase enzyme solution* was prepared by sonicating 0.625 g of rat intestinal acetone powder in 50 mL of assay buffer II at RT, followed by vigorous mixing at 800 rpm for 20 min at RT. The mixture was centrifuged at 14,000 rpm for 30 min at 4 °C (Beckman Coulter Avanti J-25i high-speed centrifuge), and the supernatant (working enzyme solution) was stored in aliquots in the dark at 4 °C until use. The working α-glucosidase solution can be refrigerated for storage for up to 3 months without any appreciable decline in enzyme activity. The working enzyme solution cannot be frozen as freezing inactivates the enzyme.
  Comments: Note that the concentration of α-glucosidase in the enzyme solution is unknown. However, different volumes of the enzyme solution (10, 20 and 30 µL/well) were tested as part of the assay optimisation and 30 µL/well was optimal for the assay method described herein.
- *p-NPG substrate solution* was prepared by dissolving and diluting *p*-NPG powder in assay buffer II to obtain a working substrate solution of 5 mM.

##### α-glucosidase assay step-by-step

**Fig. 4:**
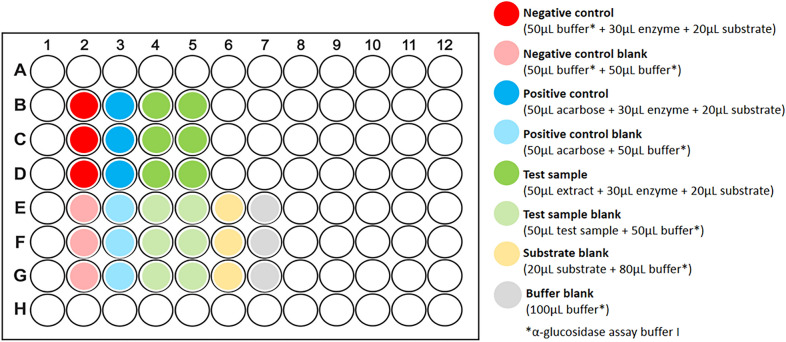
Suggested plate layout for the α-glucosidase assay

The absorbance-based α-glucosidase assay was performed in Corning^®^ Costar^®^ tissue culture-treated clear, flat-bottom 96-well plates. The assay method is described below including volumes of all test samples, controls, and assay reagents and a suggested plate layout is given in Fig. 4.

**Step 1:** Set up 96-well plate with test samples, controls, and blanks.

Firstly, assay buffer I was added to the wells for the negative control (50 µL/well) and the various blanks. Then the test samples and acarbose (50 µL/well of 1 mg/mL, final concentration 0.5 mg/mL) were added to their respective test wells and corresponding blank wells.

Comments: Assay buffer I was added to the sample blank wells and control blank wells to make their volumes and concentrations equal to the volumes and concentrations in the test wells. It was not necessary to add the substrate to the substrate blanks in Step 1 because, unlike the samples and controls which were all added at the start of the assay, the substrate was only added in Step 3 after pre-incubation. Temperature variation between outer and inner wells and increased evaporation in the outermost wells can give rise to edge effects which contribute to the degradation of assay results [96, 97]. To avoid this, only the central wells were used in the assay and the peripheral wells were excluded.

**Step 2:** Add enzyme and pre-incubate with test samples.

Using a multichannel pipette, the enzyme solution (30 µL/well) was added to all test wells and mixed by gentle pipetting. The plate was incubated in the dark at 37 °C for 10 min.

Comments: ‘Test wells’ refers to the wells containing enzyme + substrate and a test sample or control. Care must be taken not to create air bubbles and avoid foaming during mixing as aeration can denature the enzyme [98, 99]. In addition, entrapped air bubbles refract light and can therefore cause interference with absorbance measurements [47]. A temperature of 37 °C was chosen for all incubation steps in the α-glucosidase assay, as being close to body temperature, it is physiologically relevant and is optimal for mammalian enzyme activity [100].

**Step 3:** Add substrate to start reaction and incubate plate.

Using a multi-channel pipette, the substrate solution (20 µL/well of 5 mM, final concentration 1 mM) was added to all test wells and the substrate blank wells and mixed by gentle pipetting. The plate was incubated in the dark at 37 °C for 20 min.

Comments: Substrate was not added to the sample blanks, control blanks, or the buffer blanks. The plate was incubated in the microplate reader CLARIOstar^®^ microplate reader (BMG LABTECH) set at 37 °C.

**Step 4:** Measure absorbance using plate reader.

Absorbance was measured at 405 nm using the microplate reader set to 37 °C. The plate was read from the top.

#### Fluorescence assays: α-amylase and lipase

##### Preparation of herbal extract granules for testing

To prepare the samples for testing, the herbal extract granules of *Gardenia jasminoides* and *Nelumbo nucifera* (for the α-amylase assay), and the granules of *Camellia sinensis* and *Sophora japonica* (for the lipase assay) were ground to a fine powder using a mortar and pestle, and 20 mg of the ground granules were dissolved in 4 mL of distilled water containing 2% DMSO to obtain 5 mg/mL stock solutions. These were vortexed for 5 min and sonicated for 10 min to aid solubilisation, and then filtered through a Millex-HP 0.45 µm filter (Millipore). The test sample stock solutions thus prepared were diluted in assay buffer I to prepare working solutions as described below.

#### α-amylase assay

##### Preparation of buffers, reagent solutions, test samples and controls

- *α-amylase assay buffer I* (for diluting test samples and preparing the positive control acarbose) consisted of 10 mM sodium phosphate, 2.68 mM KCl, 140 mM NaCl and 1 mM CaCl_2_ (pH 6.9) and 2% DMSO.
  Comments: DMSO was used to aid the solubility of the extracts in the buffer [94, 95]. Preliminary testing confirmed that 2% DMSO (final concentration of 0.001% DMSO) had no significant effect on α-amylase activity in this assay.
- *α-amylase assay buffer II* (for diluting substrate and enzyme) consisted of 10 mM sodium phosphate, 2.68 mM KCl, 140 mM NaCl and 1 mM CaCl_2_ (pH 6.9).
- *Substrate buffer* (solvent to dissolve substrate DQ ™ starch) provided with the EnzChek™ *Ultra* Amylase assay kit, consisted of 50 mM sodium acetate buffer, pH 4.0
- *Test samples* (20 mg of herbal extract granules) were dissolved in distilled water with 2% DMSO (4 mL) to prepare a stock solution 5 mg/mL and diluted in α-amylase assay buffer I to obtain a working solution of 1.2 mg/mL (final concentration in well = 0.3 mg/mL). Samples were freshly prepared before experiments and used immediately.
- *Positive control acarbose* (20 mg) was dissolved in α-amylase assay buffer I (4 mL) to obtain a 5 mg/mL stock solution and diluted to obtain a solution of 1.2 mg/mL (final concentration of 0.3 mg/mL) immediately before experiments.
- *Negative control* (vehicle) was α-amylase assay buffer I.
- *Porcine pancreatic α-amylase* (10^7^ mU/mL) was serially diluted in α-amylase assay buffer II to obtain a working enzyme solution of 48 mU/mL.
- *BODIPY*^*®*^*FL-DQ™ starch substrate* was prepared by dissolving first in substrate buffer, then in α-amylase assay buffer II to obtain a stock solution of 1 mg/mL as per the manufacturer’s instructions. A working substrate solution of 200 µg/mL was prepared by serial dilution.

##### α-amylase assay step-by-step

A fluorescence assay for determining porcine pancreatic α-amylase activity using DQ™ starch as a substrate was performed in Nunc™ Nunclon™ delta treated, black polystyrene, flat-bottom 96-well plates using a method adapted from [45].

**Step 1:** Set up 96-well plate with test samples, controls, and blanks.

Firstly, α-amylase assay buffer I was added to the negative control wells (25 µL/well) and the various blanks. Then the test samples and acarbose (25 µL/well of 1.2 mg/mL, final concentration 0.3 mg/mL) were added to their respective test wells and their corresponding sample blank or positive control blank wells.

Comments: Assay buffer I was added to the sample blank wells and control blank wells to make their volumes and concentrations equal to the volumes and concentrations of the test wells. Assay buffer I was also the negative control (vehicle). It was not necessary to add substrate to the substrate blanks in Step 1 because, unlike the samples and controls which were all added at the start of the assay, the substrate was only introduced in Step 3 after pre-incubation. Temperature variation between outer and inner wells and increased evaporation in the outermost wells can give rise to edge effects which contribute to the degradation of assay results [96, 97]. To avoid this, only the central wells were used in the assay and the peripheral wells were excluded.

**Step 2:** Add enzyme and pre-incubate with test samples.

Using a multichannel pipette, the amylase solution (25 µL/well of 48 mU/mL, final concentration 12 mU/mL) was added to all test wells and mixed by gentle pipetting. Assay buffer I (25 µL/well) was added to all test wells to bring up the volume to 100 µL. The plate was incubated in darkness at RT for 20 min.

Comment: ‘Test wells’ refer to the wells containing enzyme + substrate and a test sample or control. Note that the well volumes were adjusted to 100 µL with the addition of buffer to maintain the total reaction mixture volumes consistent across all three assays, for convenience and calculations, and to minimise the effects of evaporation during incubation. This is not strictly necessary and adjusting the volumes (and concentrations) of enzyme, substrate, and test substances to a total of 100 µL is suggested.

**Step 3:** Add substrate to start the reaction and incubate plate.

Using a multichannel pipette, the substrate solution (25 µL of 200 µg/mL, final concentration 50 µg/mL) was added to all test wells and the substrate blank wells and mixed thoroughly by gentle pipetting. Next, the plate was incubated in the dark at RT for 20 min.

Comments: Substrate was not added to the sample blanks, control blanks, or the buffer blanks.

**Step 4:** Measure fluorescence using a plate reader.

Fluorescence was measured with the CLARIOstar^®^ microplate reader (BMG LABTECH) at excitation and emission wavelengths of 485 nm and 530 nm, respectively, over 30 min.

#### Lipase assay

##### Preparation of buffers, reagent solutions, test samples and controls

- *Lipase assay buffer I* (for diluting test samples and preparing the positive control orlistat, and the substrate) consisted of 13 mM Tris-HCl, 75 mM NaCl, 1.3 mM CaCl_2_ (pH 8.0) and 2% DMSO.
  Comments: DMSO was used to aid with the solubility of the extracts in the buffer [94, 95]. Preliminary testing confirmed that 2% DMSO (final concentration of 0.001% DMSO) had no significant effect on lipase activity in this assay.
- *Lipase assay buffer II* (for preparing porcine pancreatic lipase enzyme) consisted of 13 mM Tris-HCl, 75 mM NaCl and 1.3 mM CaCl_2_ (pH 8.0).
- *Test samples* (20 mg herbal extract granules) were dissolved in 4 mL distilled water containing 2% DMSO to prepare stock solutions of 5 mg/mL and diluted in lipase assay buffer I to 0.4 mg/mL (final concentration of 0.1 mg/mL). Samples were freshly prepared before experiments and used immediately.
- *Positive control orlistat* (25 mg) was dissolved in lipase assay buffer I (5 mL) to obtain a 5 mg/mL stock solution and diluted in lipase assay buffer II to 0.4 mg/mL (final concentration of 0.1 mg/mL) immediately before experiments.
- *Negative control* (vehicle) was the lipase assay buffer I.
- *Porcine pancreatic lipase enzyme* powder was dissolved in lipase assay buffer II to obtain a stock solution 3.5 × 10^4^ U/mL. Subsequently, the enzyme solution was serially diluted to obtain a working solution of 50 U/mL.
- *4-MUO substrate solution* was prepared by dissolving in lipase assay buffer I to obtain a working substrate solution of 0.1 mM.

##### Lipase assay step-by-step

**Fig. 5:**
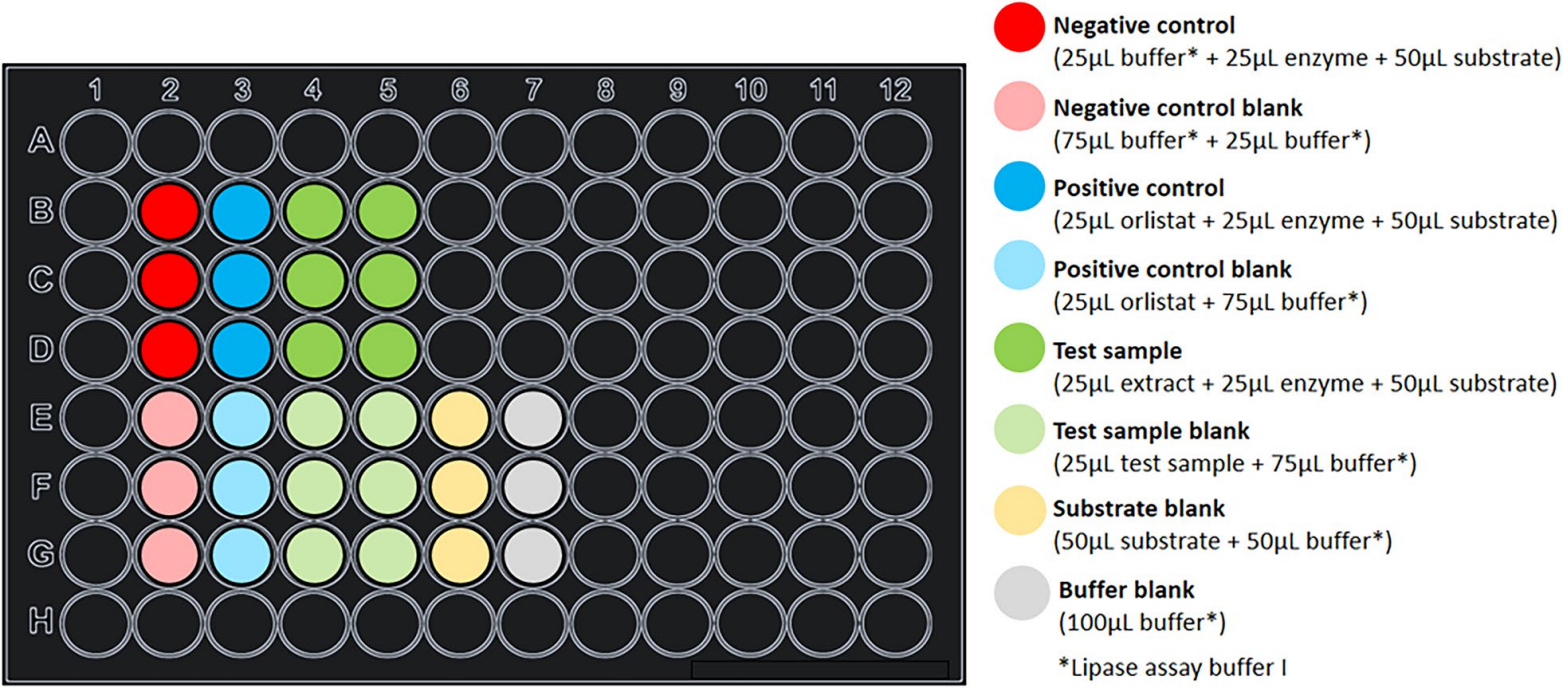
Suggested plate layout for lipase inhibition assay

The assay was adapted from methods previously described [71, 101]. The volumes of test samples, controls, and assay buffer are illustrated below using an example plate layout (Fig. 5).

**Step 1:** Set up 96-well plate with test samples, controls, and blanks.

Firstly, lipase assay buffer I was added to the negative control wells (25 µL/well) and the various blanks. Then the test samples and orlistat (25 µL/well of 0.4 mg/mL, final concentration 0.1 mg/mL) were added to their respective test wells and their corresponding sample blanks or positive control blanks.

Comments: Lipase assay buffer I was added to the sample blank wells and control blank wells to make their volumes and concentrations equal to the volumes and concentrations of the test wells. It was not necessary to add the substrate to the substrate blanks in Step 1 because, unlike the samples and controls which were all added at the start of the assay, the substrate was only introduced in Step 3 following pre-incubation. Temperature variation between outer and inner wells and increased evaporation in the outermost wells can give rise to edge effects which contribute to the degradation of assay results [96, 97]. To avoid this, only the central wells were used in the assay and the peripheral wells were excluded.

**Step 2:** Add enzyme and pre-incubate with test samples.

Using a multi-channel pipette, lipase solution (25 µL/well of 50 mU/mL, final concentration 12.5 mU/mL) was added to all test wells and mixed by gentle pipetting. Assay buffer I (25 µL/well) was added to all test wells to bring the volume up to 100 µL. The plate was incubated in darkness at RT for 5 min.

Comment: ‘Test wells’ refer to the wells containing enzyme + substrate and a test sample or control.

**Step 3:** Add substrate to start the reaction and incubate plate.

Using a multi-channel pipette, the substrate solution (50 µL of 200 µg/mL, final concentration 50 µg/mL) was added to all test wells and the substrate blanks and mixed thoroughly by gentle pipetting. Next, the plate was incubated in the dark at RT for 30 min.

Comments: Substrate was not added to the sample blanks, control blanks, or the buffer blanks.

**Step 4:** Measure fluorescence using plate reader.

Fluorescence was measured with the CLARIOstar^®^ microplate reader (BMG LABTECH) at excitation and emission wavelengths of 355 nm and 460 nm, respectively.

Comments: The lipase substrate 4-MUO had poor solubility in aqueous-based buffer. DMSO increased solubility, however, there was still some turbidity which can interfere with fluorescence measurements [62]. A possible solution would be to use a stopping reagent, centrifuge the plate, and transfer the supernatant to a new plate before fluorescence measurement.

### Calculation of results

#### Blank-correction and calculation of enzyme activity

For all three assays, enzyme activity was calculated using blank-corrected raw data. Enzyme activity was expressed as a percentage of the negative control which was normalised to 100% activity.

For comparison, raw data was blank-corrected in six different ways (Table 1) using a combination of buffer-only, substrate, and sample blanks:

**Table 1:** Formulas for blank-correction of raw absorbance data

Method	Formula for blank-correction	
	α-glucosidase assay	α-amylase and lipase assays
1	${\text{A}}_{\text{test}} - {\text{A}}_{\text{buffer blank}}$	${\text{F}}_{\text{test}} - {\text{F}}_{\text{buffer blank}}$
2	${\text{A}}_{\text{test}} - {\text{A}}_{\text{substrate blank}}$	${\text{F}}_{\text{test}} - {\text{F}}_{\text{substrate blank}}$
3	${\text{A}}_{\text{test}} - {\text{A}}_{\text{sample blank}}$	${\text{F}}_{\text{test}} - {\text{F}}_{\text{sample blank}}$
4	${\text{A}}_{\text{test}} - ( {\text{A}}_{\text{buffer blank}} {\text{ + A}}_{\text{sample blank}} )$	${\text{F}}_{\text{test}} - ( {\text{F}}_{\text{buffer blank}} {\text{ + F}}_{\text{sample blank}} )$
5	${\text{A}}_{\text{test}} - ( {\text{A}}_{\text{substrate blank}} {\text{ + A}}_{\text{sample blank}} )$	${\text{F}}_{\text{test}} - ( {\text{F}}_{\text{substrate blank}} {\text{ + F}}_{\text{sample blank}} )$
6	${\text{A}}_{\text{test}} - [\left( {{\text{A}}_{\text{substrate blank}} - {\text{A}}_{\text{buffer blank}} } \right){\text{ + A}}_{\text{sample blank}} ]$	${\text{F}}_{\text{test}} - [\left( {{\text{F}}_{\text{substrate blank}} - {\text{F}}_{\text{buffer blank}} } \right){\text{ + F}}_{\text{sample blank}} ]$

*A* and *F* refers to absorbance and fluorescence respectively

A_test_ = the absorbance of treatment wells (enzyme + substrate + plant extract), positive control wells (enzyme + substrate + positive control acarbose) and negative control wells (enzyme + substrate + vehicle)

A_buffer blank_ = the absorbance of the buffer-only blank used to determine the absorbance due only to the buffer

A_substrate blank_ = the absorbance of the substrate blank (substrate + buffer)

A_sample blank_ = the absorbance of the sample blank, positive control blank, or negative control blank

F_test_ = the fluorescence of treatment wells (enzyme + substrate + plant extract), positive control wells (enzyme + substrate + positive control acarbose) and negative control wells (enzyme + substrate + vehicle)

F_buffer blank_ = the fluorescence of the buffer-only blank used to determine the absorbance due only to the buffer

F_substrate blank_ = the fluorescence of the substrate blank (substrate + buffer)

F_sample blank_ = the fluorescence of the sample blank, positive control blank, or negative control blank (Please see note below)

Note on sample blanks: In this study, each *“test sample”* (i.e. extracts, positive control (acarbose or orlistat), and negative control (vehicle) were given their own sample blank. The sample blank for the positive control, which may also be referred to as the *“positive control blank”* contained buffer + positive control. The sample blank for the negative control, which may also be referred to as the *“negative control blank”* contained buffer + negative control (vehicle). In methods 3–6, the positive and negative controls were blank-corrected using their respective positive control *“sample”* blank and negative control *“sample”* blank

1. Raw data corrected by subtracting only the buffer-only blank: RD–BB.
2. Raw data corrected using only the substrate blank: RD − SuB.
3. Raw data corrected using dedicated sample blanks for each sample, a positive control blank, and a negative control blank (“test substance blanks” for the samples and controls): RD − SaB.
4. Raw data corrected using both the buffer-only blank, and individual sample or control blanks: RD − (BB + SaB).
5. Raw data corrected using both the substrate blank, and individual sample or control blanks: RD − (SuB + SaB).
6. Raw data corrected with the difference in absorbance or fluorescence between the substrate blank and the buffer-only blank, and the individual sample and control blanks: RD − [(SuB − BB) + SaB].

The α-glucosidase activity and inhibition were calculated from the blank-corrected absorbance data as a percentage of the negative (uninhibited) control using the following formula:

$${\text{Percentage enzyme activity = }}\frac{\text{Blank-corrected Absorbance of test well}}{\text{Blank-corrected Absorbance of negative control}} \times 1 0 0$$

$${\text{Percentage enzyme inhibition = 1}} - \left( {\frac{\text{Blank-corrected Absorbance of test well}}{\text{Blank-corrected Absorbance of negative control}}} \right) \times 1 0 0$$

The α-amylase and lipase activity and inhibition were calculated from the blank-corrected fluorescence data as a percentage of the negative (uninhibited) control using the following formula:

$${\text{Percentage enzyme activity = }}\frac{\text{Blank-corrected Fluorescence of test well}}{\text{Blank-corrected Fluorescence of negative control}} \times 1 0 0$$

$${\text{Percentage enzyme inhibition = 1}} - \left( {\frac{\text{Blank - corrected Fluorescence of test well}}{\text{Blank-corrected Fluorescence of negative control}}} \right) \times 1 0 0$$

#### Statistical analyses

One-way ANOVA and Tukey’s multiple comparison test were applied to the data to establish statistical significance of differences in results between different treatment groups (i.e. negative control, positive control, and the different extracts) as well as between the different calculation methods within the same group.

## Results and discussion

**Fig. 6:**
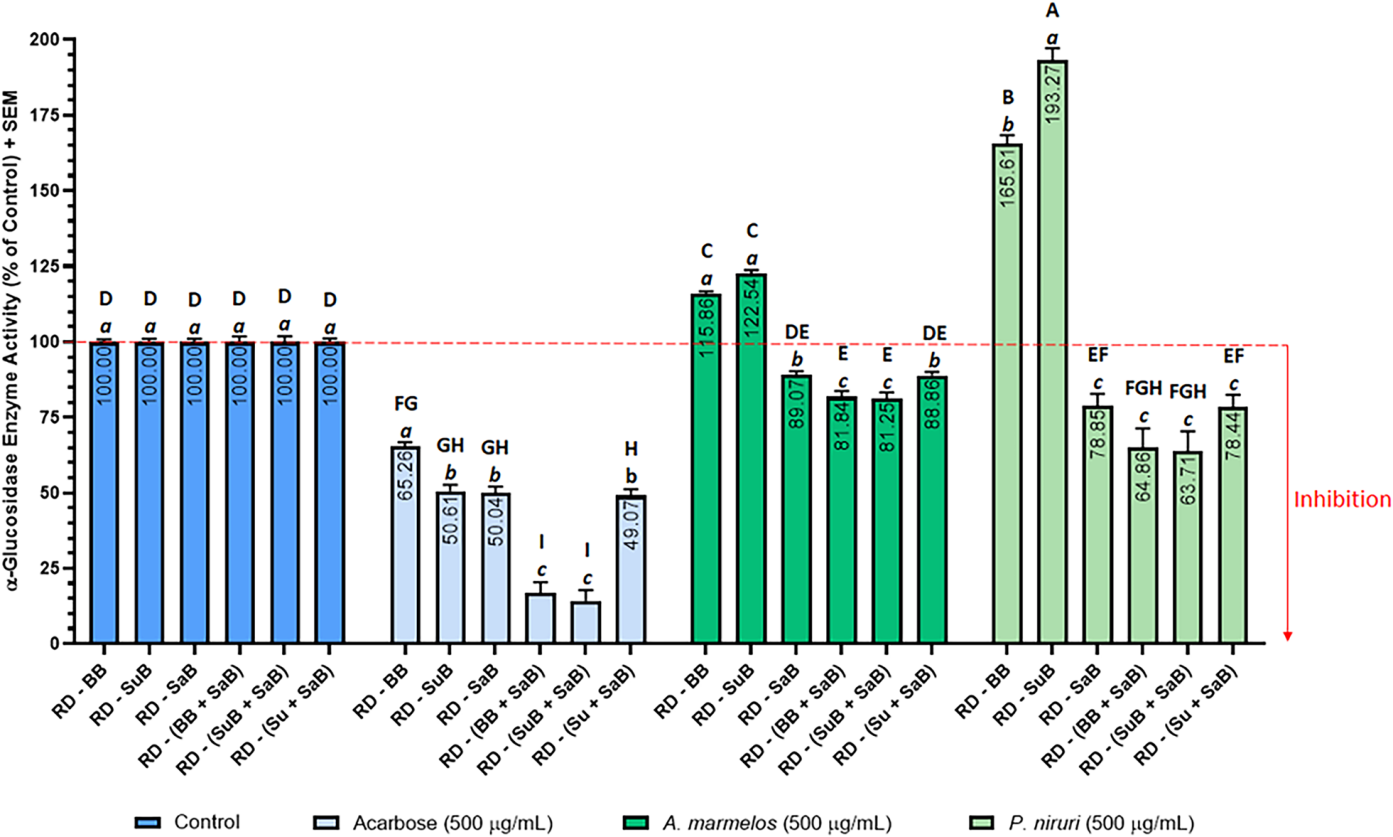
Effect of acarbose and plant extracts on rat intestinal α-glucosidase activity. Acarbose (positive control), and the *Aegle marmelos* and *Phyllanthus niruri* leaf extracts were tested at 0.5 mg/mL. The negative control (uninhibited control) was normalised to 100% activity. Data represent the mean + SEM of triplicate readings (*n *=3). Bars that do not share a letter were significantly different from each other as determined by one-way ANOVA and Tukey’s multiple comparison test (*p *< 0.05). Uppercase letters denote significance between treatment groups and lowercase letters denote significance within groups. RD: raw data, BB: buffer blank (contained buffer only), SuB: substrate blank (contained substrate + buffer), and SaB: sample blank. Sample blank composition: *A. marmelos* sample blank = *A. marmelos* extract + buffer, *P. niruri* sample blank = *P. niruri* extract + buffer, acarbose sample blank (positive control blank) = acarbose + buffer, and negative control sample blank (negative control blank) = vehicle + buffer (as the vehicle was buffer, this contained buffer only)

**Fig. 7:**
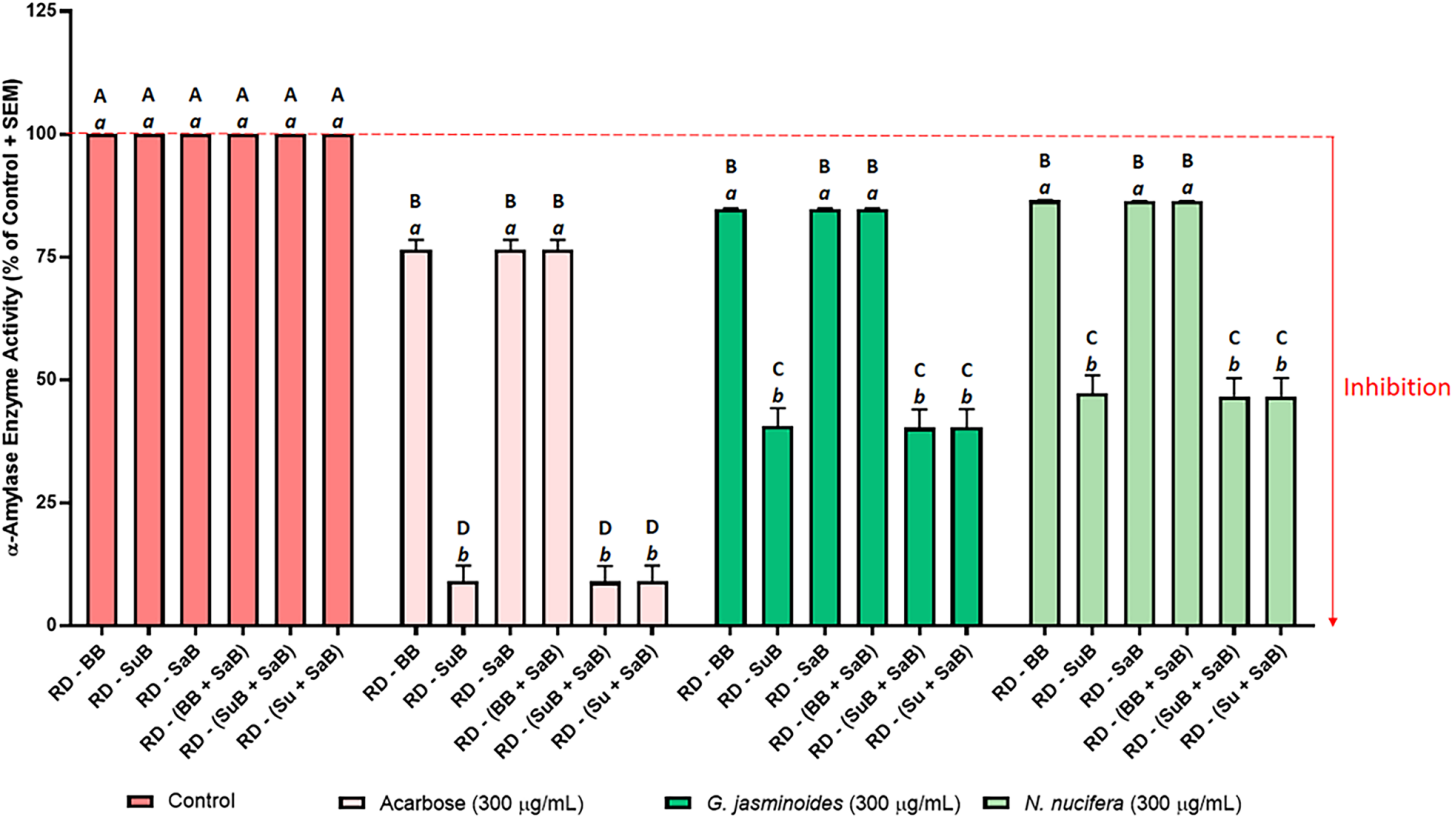
Effect of acarbose and plant extracts on porcine pancreatic α-amylase activity. Acarbose (positive control), and the *Gardenia jasminoides* and *Nelumbo nucifera* extracts were tested at 0.3 mg/mL. The negative control (uninhibited control) was normalised to 100% activity. Data represent the mean + SEM of readings from three independent experiments. Bars that do not share a letter were significantly different from each other as determined by one-way ANOVA and Tukey’s multiple comparison test (*p *< 0.05). Uppercase letters denotes significance between treatment groups and lowercase letters denote significance within groups. RD: raw data, BB: buffer blank (contained buffer only), SuB: substrate blank (contained substrate + buffer), and SaB: sample blank. Sample blank composition: *G. jasminoides* sample blank = *G. jasminoides* extract + buffer, *N. nucifera* sample blank = *N. nucifera* extract + buffer, acarbose sample blank (positive control blank) = acarbose + buffer, and control sample blank (negative control blank) = vehicle + buffer (as the vehicle was buffer, this contained buffer only)

**Fig. 8:**
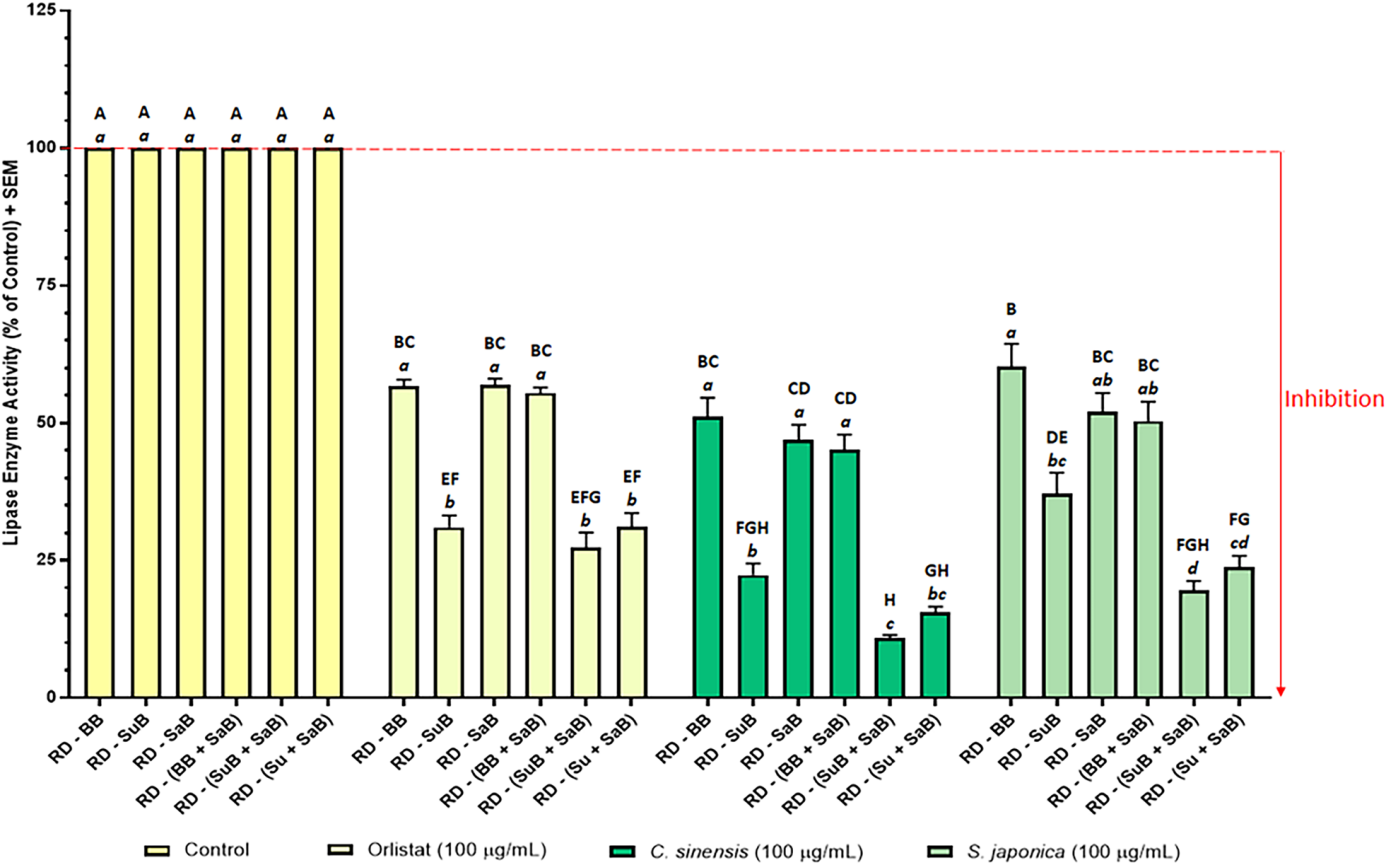
Effect of orlistat and plant extracts on porcine pancreatic lipase activity. Orlistat (positive control), and the *Camellia sinensis* and *Sophora japonica* extracts were tested at 0.1 mg/mL. The negative control (uninhibited control) was normalised to 100% activity. Data represent the mean + SEM of readings from three independent experiments. Bars that do not share a letter were significantly different from each other as determined by one-way ANOVA and Tukey’s multiple comparison test (*p *< 0.05). Uppercase letters denote significance between treatment groups and lowercase letters denotes significance within groups. RD: raw data, BB: buffer blank (contained buffer only), SuB: substrate blank (contained substrate + buffer), and SaB: sample blank. Sample blank composition: *C. sinensis* sample blank = *C. sinensis* extract + buffer, *S. japonica* sample blank = *S. japonica* extract + buffer, orlistat sample blank (positive control blank) = orlistat + buffer, and control sample blank (negative control blank) = vehicle + buffer (as the vehicle was buffer, this contained buffer only)

In all three assays, the positive controls—acarbose for α-glucosidase (Fig. 6) and α-amylase (Fig. 7), and orlistat for lipase (Fig. 8)—caused a significant reduction in enzyme activity when compared with the uninhibited control. This is as expected and provided validation of the assay methods and confirmed the suitability of the assays for their intended purpose [102].

Blank-correction using just the buffer blank (RD − BB) yielded high enzyme activity, and therefore low enzyme inhibition results in all three assays (Figs. 6, 7 and 8). In the α-glucosidase assay, RD − BB exhibited the highest enzyme activity value for acarbose, and the second highest enzyme activity values for *A. marmelos* and *P. niruri.* Similarly, in the lipase assay, RD − BB yielded the highest enzyme activity for *C. sinensis* and *S. japonica,* and the second highest for orlistat. In the α-amylase assay, RD − BB gave very high enzyme activity, however, these enzyme activity values were almost identical to RD − SaB and RD − (BB + SaB).

**Fig. 9:**
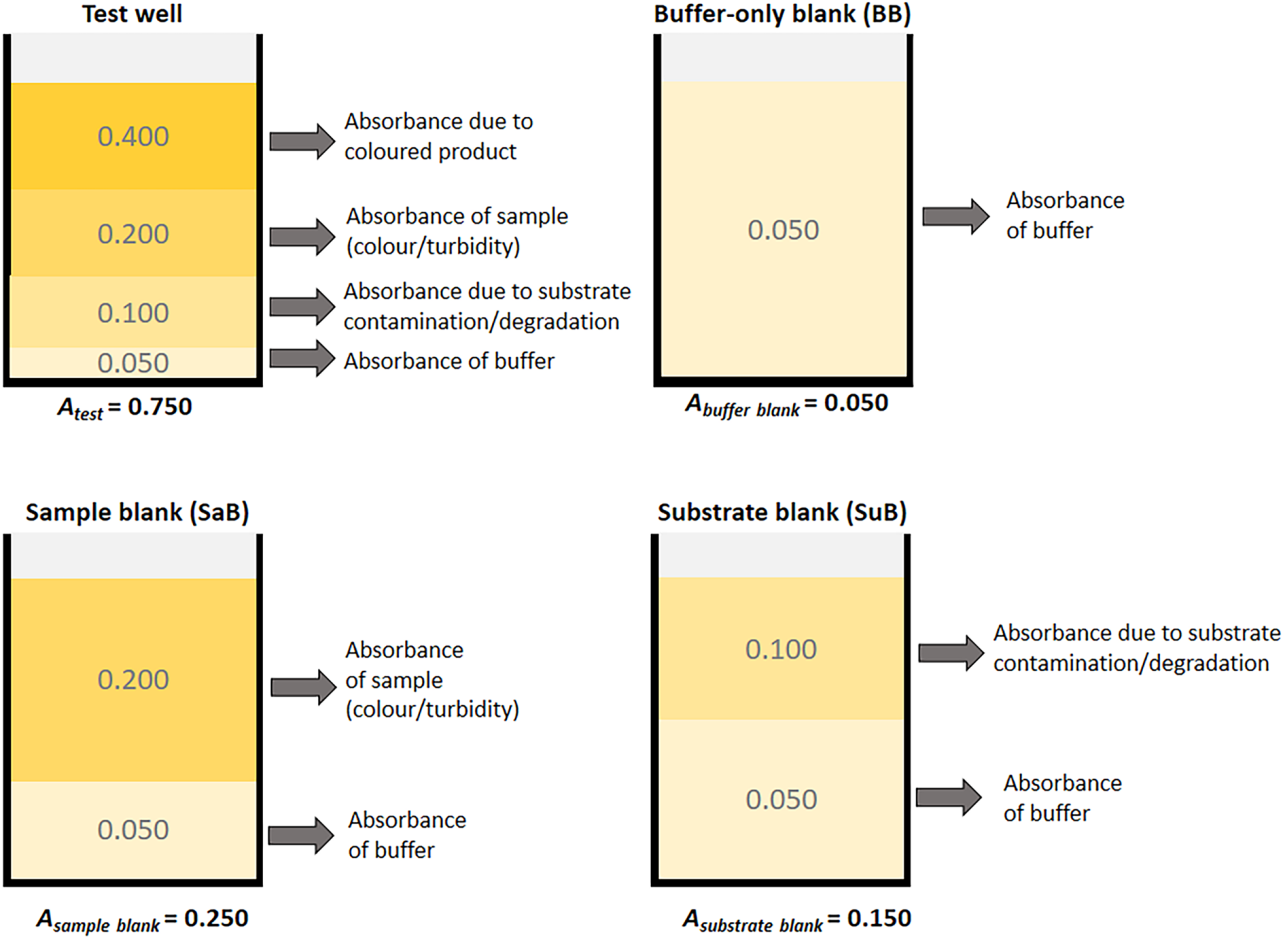
Components contributing to absorbance. This figure illustrates the various components contributing to the absorbance in the test wells, buffer-only blank, sample blank, and substrate blank wells. This also applies to fluorescence assays. Absorbance values in this figure are for illustrative purposes only (not based on real values)

Buffer blanks are routinely used for baseline correction and account for the background absorbance or fluorescence of the assay buffer. However, if no substrate blank or sample blanks are included, this method (RD − BB) does not account for interference from the substrate or samples. Therefore, in RD − BB, the background absorbance (or fluorescence) of the substrate and the samples, which caused an additive effect on the test well measurements were uncorrected and resulted in misleadingly high enzyme activity results. This is also illustrated in Fig. 9 which shows the components contributing to the total absorbance (or fluorescence) of a test well, buffer blank, sample blank, and substrate blank. Table 2 provides an example of how the different blanking methods used in this study changed the blank-corrected absorbance (or fluorescence) of the test well.

**Table 2:** Effect of blank-correction methods on the test well absorbance values in Fig. 9

Method	Formula	Calculation	Blank-corrected absorbance
1	RD − BB	0.750 − 0.050	0.700
2	RD − SuB	0.750 − 0.150	0.600
3	RD − SaB	0.750 − 0.250	0.500
4	RD − (BB + SaB)	0.750 − (0.050 + 0.250)	0.450
5	RD − (SuB + SaB)	0.750 − (0.150 + 0.250)	0.350
6	RD − [(SuB − BB) + SaB)]	0.750 − [(0.150 − 0.050) + 0.250]	0.400

RD: raw data (A_test_); BB: buffer blank; SuB: substrate blank, SaB: sample blank

**Fig. 10:**
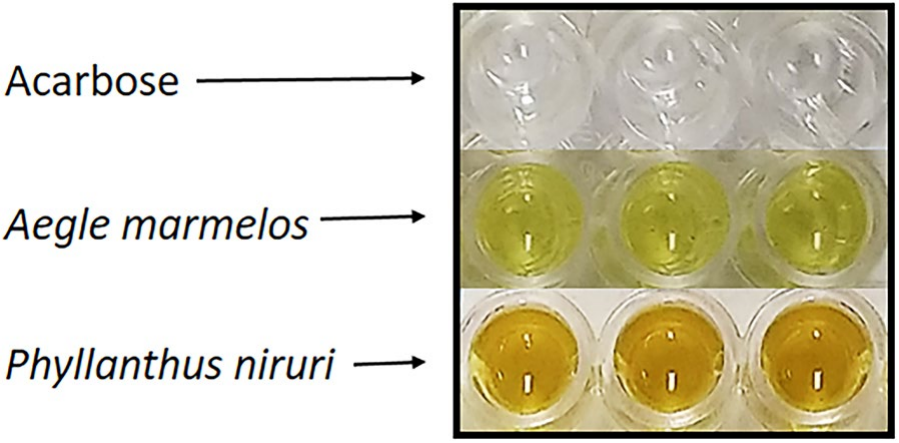
Acarbose, *A. marmelos* and *P. niruri* extracts at 0.5 mg/mL in a clear 96-well plate

In the α-glucosidase assay (Fig. 6), enzyme inhibition by the two plant extracts *A. marmelos* and *P. niruri* was only observed when a sample blank was included [RD − SaB, RD − (BB + SaB), RD − (SuB + SaB), and RD − (Su + SaB)] to offset the interference due to the colour of the extracts (Fig. 10). When results were calculated without a sample blank (RD − BB and RD − SuB), *A. marmelos* and *P. niruri* appeared to promote enzyme activity. This apparent increase in enzyme activity can be attributed to the colour of the two extracts contributing an additive effect to the absorbance of the test wells resulting in an over-estimation of enzyme activity. Note that the interferences due to sample colour may be minimised by diluting the samples. However, if the sample is too dilute for inhibitory activity to be detected, screening samples at low concentrations increases the risk of missing potential *hits*.

In contrast to the plant extracts, acarbose inhibited α-glucosidase significantly regardless of whether or not a sample blank was included in the blank-correction. Acarbose was colourless and dissolved completely in α-glucosidase assay buffer I to give a clear, colourless solution (Fig. 10). Hence, any spectral interference due to the background absorbance of acarbose was negligible and not correcting this background absorbance did not affect the final results as much as the highly coloured extracts which had high background absorbances (sample blank absorbance).

Figures 7 and 8 illustrate the effects of the six blanking methods on the inhibition of α-amylase and lipase. Regardless of the blanking method used, all test samples and positive controls (acarbose for α-amylase, and orlistat for lipase) showed significant inhibition of their respective enzymes. This contrasted with the absorbance-based α-glucosidase assay, where not including a sample blank gave enzyme activity > 100% for the two plant extracts. The reason for this was that the colours of the extracts used in the α-glucosidase assay had high background absorbance values relative to the absorbance of the test wells and therefore contributed a larger error to the final results. Correcting this large error by subtracting the sample blanks caused a larger reduction in the final results. On the other hand, the background fluorescence of the samples in the amylase and lipase assays were much smaller relative to the fluorescence of the test wells, and therefore only contributed a smaller error to the final results.

Although the sample blank did not influence the enzyme activity values in the fluorescence assays, the inclusion or omission of a substrate blank significantly impacted the final enzyme activity values in both the α-amylase (Fig. 7) and lipases assays (Fig. 8). The enzyme activity results within each group (each of the positive controls and plant extracts) separated into two tiers depending on whether a substrate blank had been included in the blank-correction: the enzyme activity of all groups were significantly higher when a substrate blank was not included [RD − BB, RD − SaB, and RD − (BB + SaB)] than when a substrate blank was included (RD − SuB, RD − (SuB + SaB), and RD − (Su + SaB). This was attributed to the high background autofluorescence of the substrate (157,700 for α-amylase substrate, and 35,632 for lipase substrate) which contributed a proportionally larger error to the final results (e.g. for *G. jasminoides,* F_test_ = 193222, F_sample blank_ = 179 vs F_substrate blank_ = 171765). Subtracting the high background autofluorescence of the substrate (substrate blank) proportionally reduced the blank-corrected fluorescence of the test wells (193,222 − 171,765 = 21,457 when substrate blank was included versus 193,222 − 179 = 193,044 when the sample blank was included) and therefore reduced the final calculated enzyme activity values. In the α-glucosidase assay, the substrate had a comparatively smaller effect on the enzyme activity results because the background absorbance due to the substrate was lower when compared to the raw absorbance data of the test wells. These results indicated that it is crucial to include a substrate blank to avoid the risk of missing potential enzyme inhibitory activity in drug candidates due to the overestimation of enzyme activity.

Subtracting two blanks [RD − (BB + SaB) and RD − (SuB + SaB)] generally resulted in lower enzyme activity than for the methods that only subtracted one blank. Note that both the substrate blank and the sample blank contains a certain volume of buffer (Fig. 9). Therefore, the absorbance or fluorescence of these blanks was in part, due to the buffer, and only a proportion of the absorbance was due to the substrate or sample. If two blanks were subtracted during blank-correction, as in the case with RD − (BB + SaB) and RD − (SuB + SaB), this *“hidden”* background due to the buffer would be subtracted twice, resulting in *“double*-*blanking.”* In the literature, some studies have combined sample and substrate in a single blank [65, 79, 80]. This approach prevents double-blanking the buffer as only one blank (containing buffer + sample + substrate) is subtracted from the raw data. However, this must be carried out with caution as unexpected results may occur when the substrate interacts with unknown constituents in the sample; especially in crude natural product extracts which contain complex mixtures of compounds and are a notorious, complicated biological matrix. Another drawback to this approach is that the substrate must be added to each sample blank and this would therefore require larger amounts of substrate per assay which adds to the cost of the assay. In method six [RD − (Su + SaB)], the buffer blank was subtracted from the substrate blank to obtain the absorbance or fluorescence contributed by the substrate only (Su = SuB − BB) and this value was subtracted from the raw data along with the sample blank [RD − (Su + SaB)]. This allowed for the correction of interference due to the sample and the substrate, without double-blanking the buffer, and without adding the substrate to each sample blank and any unexpected artefacts which may arise from doing so. Therefore, [RD − (Su + SaB)] was the most appropriate blanking method for all three assays.

## Conclusion

This is the first study to investigate the effects of using different blanks and blanking methods on the results of enzyme assays, and to provide comprehensive, *step*-*by*-*step* protocols for α-amylase, α-glucosidase, and lipase-inhibition assays that can be performed in 96-well format, in a simple, fast, and resource-efficient manner, with clear instructions for blank-correction and calculation of results.

The type of blanks and blank-correction methods employed in the assays had a significant effect on the final, calculated enzyme activity (and inhibition) values in all three assays. The importance of including a sample blank when testing highly coloured samples, as well as the relevance of a substrate blank, and avoiding errors due to unintended double-blanking were highlighted in the results. Depending on the blank(s) used in the blank-correction of raw data, variation in the final calculated results can lead to either an over-estimation or under-estimation of the calculated enzyme activity. Not accounting for interferences due to the colour of the natural product extracts can result in misleadingly high enzyme activity values which underestimates the bioactivity of the target sample. Therefore, potential enzyme inhibitors can be inadvertently overlooked resulting in missed opportunities in the drug discovery process.

The variation in the final calculated results demonstrated that the same set of raw data can produce different results depending on the blank-correction method. This emphasises the importance of standardising the use of blanks and the reporting of blank-correction procedures in published literature in order to enhance the reproducibility of results and prevent misleading results in not only enzyme assays, but also in other spectrometry-based assays involving natural product extracts.

Out of the methods tested, the sixth blanking method [RD − (Su + SaB)] adequately accounted for interferences due to the background absorbance/fluorescence of both the substrate and sample without double blanking, and using lower volumes of substrate, and is therefore recommended for the blank-correction of raw data in enzyme assays.

## Data Availability

The datasets used during the current study are available from the corresponding author on reasonable request.

## Abbreviations

4-MU: 4-Methylumbelliferone
4-MUO: 4-Methylumbelliferyl oleate
*A*_*subscript*_: Absorbance
BB: Buffer blank
BODIPY^®^: Boron-dipyrromethene
BODIPY^®^FL-DQ™: Boron-dipyrromethene fluorescent dye quenching
Cat. No.: Catalogue number
DMSO: Dimethyl sulfoxide
DQ™: Dye quenching
DQ™ starch: Dye quenching starch
EC: Enzyme commission number
*F*_*subscript*_: Fluorescence
PBS: Phosphate buffered saline
*p*NPG: *p*-Nitrophenyl-*α*-d-glucopyranoside
RD: Raw data
RT: Room temperature (25 °C)
SaB: Sample blank
SuB: Substrate blank
